# Histone modification cross-talk: analytical tools and molecular mechanisms

**DOI:** 10.1042/BCJ20250115

**Published:** 2026-04-17

**Authors:** Jennifer Jiang, Sarah DuBois-Coyne, Eunju Nam, Samuel D. Whedon, Kwangwoon Lee, Philip A. Cole

**Affiliations:** 1 Division of Genetics, Department of Medicine, Brigham and Women’s Hospital, Boston, MA 02115, U.S.A.; 2 Department of Biological Chemistry and Molecular Pharmacology, Harvard Medical School, Boston, MA 02115, U.S.A.; 3 Department of Physics and Chemistry, Daegu Gyeongbuk Institute of Science and Technology (DGIST), Daegu 42988, Republic of Korea; 4 Division of Medicinal Chemistry and Pharmacognosy, The Ohio State University, Columbus, Ohio 43210, U.S.A.

**Keywords:** chromatin, histone, nucleosome

## Abstract

Chromatin function emerges from combinatorial patterns of histone post-translational modifications (PTMs) that are read, written, and erased by dedicated enzymes. Over the past 30 years, increasing evidence suggests that specific histone PTMs or combinations of PTMs influence one another, constituting epigenetic cross-talk that shapes chromatin structure, protein–protein interactions, and catalytic efficiency of nucleosome-targeting enzymes. Here, we summarize mechanistic and methodological advances that enable rigorous interrogation of histone PTM interplay. We highlight selected nucleosome engineering strategies that build precisely modified substrates to test *in vitro*, proteomic pipelines that preserve combinatorial information, and omics technology that can globally profile integrated chromatin regulatory events in cells and tissues. Furthermore, we survey multivalent reader modules and engineered biosensors that report combinatorial marks in nucleosomes and living cells. Representative case studies illustrate how defined PTMs modulate catalytic parameters of writer and eraser complexes, including lysine methyltransferases, demethylases, acetyltransferases, and deacetylases, focusing on cross-talk with histone H3 N-terminal tail marks. These include the role of H3K9me2/3 and K14ac in directing propagation of H3K9me3, the role of H3K4me1/2 and K14ac in slowing H3K4 demethylation, the role of H3K4me2/3 in directing H3K9 acetylation, and the role of H3K36 methylation in directing deacetylation of H3 and H4. The substrates for these case studies include both mononucleosomes and nucleosome arrays. These examples illustrate the principle of epigenetic cross-talk, namely, that specific combinatorial PTMs can affect enzymes and alter local biochemistry.

## Introduction

Eukaryotic nuclear DNA is organized into chromatin, which exists in dynamic open (euchromatin) and compact (heterochromatin) states [1]. DNA in euchromatin is more accessible to transcriptional machinery, whereas DNA in heterochromatin tends to be transcriptionally silenced [2,3]. The fundamental unit of chromatin is the nucleosome, composed of 147 base pairs of double-stranded DNA wrapped around a histone octamer containing two copies each of histones H2A, H2B, H3, and H4 [4]. Nucleosome stability is driven in part by electrostatic interactions between the negatively charged DNA backbone and the positively charged histone core and N-terminal tail regions [5]. Chromatin structure can be modulated by post-translational modifications (PTMs) on histones, also known as histone marks. The most well-studied PTMs on histones are reversible Lys acetylation, Lys methylation, Lys ubiquitination, Arg methylation, and Ser/Thr phosphorylation [6,7]. Over the past two decades, a much wider range of histone PTMs has been discovered, including Lys acylations like propionylation [8], lactylation [9], β-hydroxybutyrylation [10], crotonylation [11], among other PTMs. Individual histone PTMs can induce local structural effects, such as altering DNA–histone contacts or modulating protein–protein interactions [12]. For example, Lys acetylation can suppress salt bridge interactions between unmodified Lys side chains and the DNA phosphate backbone in a nucleosome [13]. Histone Lys acetylation and other acylations reduce basic charges and can also recruit proteins through their bromodomains or YEATS domains [14,15]. In contrast, Lys methylation preserves charge but coordinates binding interactions through domains such as Tudor domains and PHD fingers [16]. On the other hand, the negative charges introduced by phosphorylation of Ser, Thr, and Tyr residues, cause histone tails to dissociate from DNA and act as docking sites for phosphorylation readers like 14-3-3 proteins [17,18].

Given the importance of histone modifications in modulating chromatin structure and gene regulation in normal and disease states, there have been intensive efforts to try to understand the enzymes that install histone PTMs (also known as writers), remove PTMs (also known as erasers) and the proteins that bind histone PTMs (also known as readers). There are numerous acetyltransferases (HATs or KATs), methyltransferases (KMTs), ubiquitin E3 ligases, and kinases that have been subject to study in the context of histones and chromatin. Moreover, there are a sizable set of erasers including deacetylases (HDACs), methyl-Lys demethylases (KDMs), phosphatases, and deubiquitinases. Many regulatory PTMs cluster on the accessible N-terminal histone tails—most prominently on H3 at K4, K9, K14, K18, K23, K27, and K36. These residues lie outside of the nucleosome core and act as hotspots for combinatorial cross-talk, where one modification can shape the addition, removal, or recognition of another [19].

## Chromatin and PTM cross-talk

The diversity and wide distribution of histone PTMs led to the hypothesis that their combinations might constitute a “histone code” that epigenetically regulates gene activity. In this view, PTMs can exhibit cross-talk—where a modification at one histone site influences the addition, removal, or recognition of another. Cross-talk can modulate how writers and erasers recognize or process their substrates, and how readers bind. While it does not seem that the PTM pattern has the precision of the genetic code that refers to DNA sequence encoding specific amino acids in proteins, there are an increasing number of cases reported where particular combinations of histone PTMs result in biochemical impacts.

### Methods for studying histone PTM cross-talk

To probe the complexity of histone PTM cross-talk, researchers have developed a suite of nucleosome engineering techniques that allow precise installation, visualization, and manipulation of PTMs. Several techniques and methods will be discussed here, namely nucleosome *in vitro* reconstitution and engineering, mass spectrometry analysis and PTM profiling, and designer PTM probe binding proteins.

#### Nucleosome reconstitution and engineering

Nucleosome engineering allows the construction of nucleosomes with defined and combinatorial PTMs, enabling controlled studies of how specific PTM patterns influence each other and contribute to histone cross-talk. The foundation of nucleosome engineering was laid in the 1970s to 1990s, when basic *in vitro* reconstitution techniques were developed. One of the earliest and most enduring methods involved salt gradient dialysis, in which purified histones and DNA were assembled into nucleosomes under decreasing ionic strength, mimicking the conditions of chromatin compaction in the cell [20]. In the 1970s, researchers commonly bulk-extracted histones from calf thymus or chicken erythrocytes. Even though these histones carried endogenous PTMs, histone bulk extraction methods can suffer from heterogeneity and poor scalability. The development of molecular biology and recombinant protein expression technology in the 1980s allowed researchers to produce individual histone proteins in *Escherichia coli*. A major advancement in the 2000s was the application of the Widom 601 DNA sequence, identified through SELEX screening [21]. Its high affinity for histone octamers, which are typically reconstituted from bacterially expressed *Xenopus laevis* histones, enabled the generation of homogeneously positioned nucleosomes [22]. With the ability to construct homogeneously positioned nucleosomes *in vitro*, the focus of the field shifted to probing specific PTMs [23]. To study the effects of PTM on signaling pathways, enzyme activity, and ultimately epigenetics, tools like site-specifically modified nucleosomes, asymmetric nucleosomes and semisynthetic strategies have been developed.

#### Semisynthetic and site-specifically modified nucleosomes

Several chemical/synthetic techniques have been used to generate site-specifically modified nucleosomes for studying histone PTM cross-talk. These methods permit the generation of homogeneously modified histones that are generally not accessible by writer enzyme-catalyzed PTMs. Installations of large PTMs, such as ubiquitin and ubiquitin-like modifiers (UBLs), present distinct chemical and architectural challenges compared with small PTMs. These large PTMs introduce substantial steric bulk, require formation of isopeptide linkages to lysine side chains, and often engage in multivalent interactions that are sensitive to linkage geometry and flexibility [24]. Given the variety of histone PTMs, specialized semisynthetic strategies have been developed to install modifiers of any size with sufficient chemical fidelity and homogeneity to enable mechanistic studies of PTM cross-talk.

##### Native chemical ligation, expressed protein ligation, and cysteine conjugation

Native chemical ligation (NCL) and expressed protein ligation (EPL) have been used to generate full-length proteins bearing site-specific PTMs [25–29]. Both ligation reactions rely on an equilibrium transthioesterification between a C-terminal peptide thioester and a 1,2-aminothiol like an N-terminal cysteine. Following formation of a thioester between the peptide and the aminothiol, an S-to-N acyl shift occurs through a cyclic intermediate (usually 5-membered), resulting in a stable, native amide linkage. While NCL and EPL are increasingly used interchangeably, NCL most commonly refers to ligations between synthetic peptide segments, which allows installation of defined PTMs during the peptide synthesis. By contrast, EPL typically makes use of an expressed protein fragment with either an N-terminal cysteine or a C-terminal thioester, the latter is generally obtained from a protein-intein fusion that undergoes thiolysis to yield the C-terminal thioester [30].

**Figure 1:**
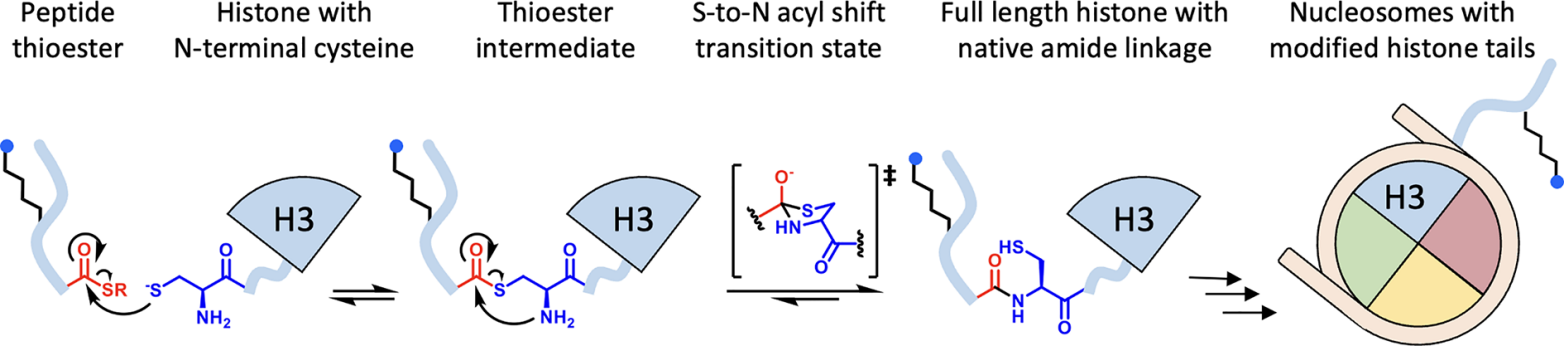
Expressed protein ligation and Native chemical ligation The mechanism of NCL and EPL begins by equilibrium transthioesterification of the N-terminal cysteine thiol and C-terminal peptide thioester (left), which is followed by an S-to-N acyl shift made more favorable by the 5-membered ring transition state (center). The reaction is rendered functionally irreversible by collapse of the tetrahedral intermediate into the native amide linkage (right) [27–29]. Expressed peptide thioesters can be obtained from a peptide-intein fusion construct, which undergoes thiolysis to afford a C-terminal thioester. Synthetic peptide thioesters can be prepared by solid phase peptide synthesis using a variety of chemical linkages to the solid phase resin. These undergo chemical activation of the C-terminus, followed by thioesterification.

EPL has been used to form native lysine isopeptide linkages between ubiquitin and histones by first generating ubiquitin C-terminal thioesters and then ligating them to histone peptides bearing lysine side chains modified with ligation auxiliaries or cysteine surrogates [30,31] (Figure 1). Following the reaction between ubiquitin thioester and lysine sidechain aminothiol a truly native isopeptide bond requires removal of the ligation auxiliary [32] (Figure 1). These approaches have enabled the production of chemically homogeneous histones bearing native H2BK120Ub or H2AK119Ub, which have been instrumental in elucidating ubiquitin-dependent allosteric activation, substrate recognition, and cross-talk mechanisms in chromatin-modifying enzymes [33].

In addition to EPL [34], other chemical biology tools have expanded the scope of histone modification research. Cysteine is rare in histone sequences, thus mutagenic installation of Cys residues followed by covalent Cys alkylation has facilitated the introduction of numerous PTM mimics [35–37]. Conversion of cysteine to dehydroalanine further expands the palette of chemical methods for derivatizing histone proteins [38]. Cysteine-based conjugation strategies also provide an alternative route for installing large PTMs like ubiquitin and Small Ubiquitin-like Modifier proteins (SUMO). This can be accomplished by mutagenic installation of a C-terminal cysteine on ubiquitin, followed by alkylation of both cysteine mutants with a reagent like 1,3-dichloroacetone [39]. Alternatively, alkylation can be used to install an EPL auxiliary at the target histone K-to-C mutation, followed by conventional reaction with a C-terminal ubiquitin thioester [40]. Finally, alkylation of the histone cysteine can be used to install an aldehyde, with reagents like chloroacetaldehyde, which can undergo reductive amination with a ubiquitin C-terminal hydrazide [41]. Although these linkages differ from native isopeptide bonds in length and flexibility, such ubiquitin mimics frequently recapitulate key biochemical properties of native ubiquitination, including stimulation of enzymatic activity and engagement by ubiquitin reader domains, making them useful for functional assays and screening applications [42]. However, because subtle geometric differences can influence higher-order interactions, cysteine-based conjugates can have limitations for high-resolution structural or mechanistic studies.

##### Sortase-mediated ligation

**Figure 2:**
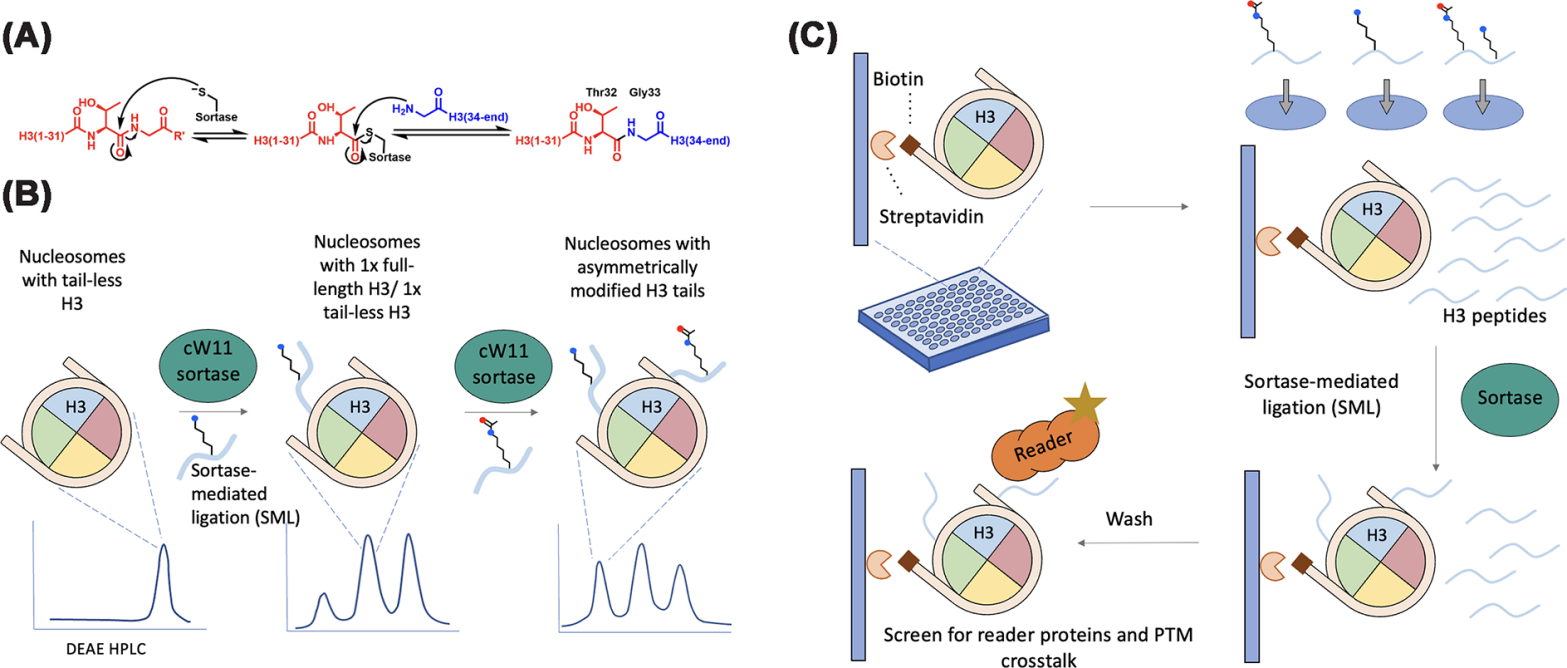
Sortase-mediated ligation: mechanism and applications (**A**) Mechanism of the sortase-mediated transpeptidation reaction applied to the semisynthesis of histone H3. (**B**) Generation of asymmetric nucleosomes by cW11 SML. Nucleosomes containing tail-less H3 (left) undergo SML with cW11 sortase and a synthetic, modified H3 tail peptide, yielding nucleosomes in which one H3 copy is full-length while the second remains tail-less (middle). A second ligation with a differently modified H3 tail peptide produces nucleosomes bearing two distinct H3 tails on the same octamer (right). Representative DEAE HPLC chromatograms below each schematic illustrate the separation and purification of the intermediate and final nucleosome species. (**C**) High-throughput platform for probing histone-reader cross-talk by SML of modified H3 tails onto immobilized nucleosomes. Top left: Biotinylated, tailless nucleosomes are tethered to streptavidin-coated wells of a 96-well microplate; Top right: Chemically defined H3 tail peptides, each bearing specific PTMs, and an LPATG depsipeptide handle, are arrayed across wells; Middle right: In each well, sortase A catalyzes SML, covalently attaching the modified H3 tails onto the nucleosome cores, generating a library of nucleosome variants in parallel; Bottom left: Following rigorous washing to remove unreacted peptides and enzyme, fluorescently tagged reader proteins (e.g., eCFP-HP1β) are added. Binding signals reveal modification-dependent affinity and enable systematic discovery of positive or negative histone PTM cross-talk, such as the enhanced recognition of H3K9me2 when adjacent H3S28 is phosphorylated.

The utility of sortase enzymes in protein semisynthesis is a powerful tool and it has opened new possibilities for site-specific incorporation of PTMs in nucleosomes (Figure 2). Sortase A (SrtA), a bacterial cysteine transpeptidase, catalyzes a transpeptidation reaction. SrtA cleaves the peptide bond between the Thr and Gly of a LPXTG motif and in a concerted fashion forms a new amide linkage with an N-terminal oligoglycine, which enables peptide ligation to proteins with regiospecificity. Sortase enzymes have been engineered for installing peptide tails carrying specific PTMs onto histones. In 2011, Piotukh and colleagues used phage display-based directed evolution to generate SrtA mutant F40 with altered substrate preferences that could recognize non-canonical motifs beyond the native LPXTG, to include H3(29–33), APATG, in the N-terminal tail [43]. This work expanded the enzyme’s ligase substrate scope, enabling its use in traceless histone semisynthesis, where maintaining native tail sequences is critical for preserving biological function.

In 2018, F40 sortase-mediated ligation (SML) was used to generate nucleosome substrates containing H3K4me2 and assorted H3 acetyl-Lys modifications [44], enhancing peptide ligation from ∼40% to ∼90% using a depsipeptide linkage between T32 and G33 of H3 tail peptides, which renders the first step of the sortase reaction with peptide irreversible [45]. This method has been extended with an engineered sortase that can generate modified H2B [46].

Recent work by Xiao and colleagues has significantly advanced SML technology for studying H4 PTMs [47]. In their 2023 study, they developed a semisynthetic approach to generate histone H4 with specific PTMs, including methylation, acetylation, and dual modifications using an engineered sortase variant, enabling the assembly of nucleosomes with multivalent H4 modifications [47]. In 2024, they improved the irreversibility and yield of SML reactions by incorporating sarkosyl, which likely traps ligated products in micelles, preventing reverse reactions and driving the reaction to completion, achieving milligram-scale synthesis, up to 98% conversion, in one-pot conditions [48]. These modified-H4 proteins can later be incorporated into nucleosomes to realize cross-talk studies.

In Bierlmeier et al., the authors introduced a novel “ligation site switching” strategy to overcome the limitations of traditional SML, which typically restricts assembly to two fragments due to the reversibility of the reaction caused by the existing recognition motif even after reaction [50]. To solve this problem, they engineered a switchable sortase substrate—using a disulfide-protected cystine (Cys(StBu)) in place of leucine to toggle the LPxTG motif between a leucine isostere (active state) and reducing it into cysteine (inactive state). In addition, they developed a photocaged N-terminal glycine on the peptide fragment, which can be activated by UV. This allows temporally controlled, step-wise synthesis of multiple peptide fragments into a single peptide containing bivalent modification mimicking H3K4me3 and H4Ac5 (K5, K8, K12, K16, and K20). The technology has the potential to be adapted to synthesize nucleosomes with both modified H3 and modified H4.

SML provides an enzyme-catalyzed route to install large protein modifiers under mild conditions and has been adapted for ubiquitin and UBL conjugation in chromatin contexts. By engineering sortase recognition motifs into histones and using modified ubiquitin or UBL substrates bearing N-terminal oligoglycine sequences, large modifiers can be site-specifically attached without the need for denaturing conditions or intein chemistry [49]. Advances in sortase engineering, including cyclization and altered substrate specificity, have improved catalytic efficiency and reduced steric constraints, enabling modification of histones within nucleosomes or higher-order assemblies. While sortase-mediated approaches introduce non-native junctions and impose sequence constraints, they offer a complementary strategy for installing large PTMs on substrates that are inaccessible to EPL-based methods.

Although SML techniques have shown robust performance *in vitro*, their adaptation for *in cellulo* applications—such as intracellular histone editing—has remained limited. In 2022, Yang et al. developed a method for adding H3 modifications in living cells using sortase-mediated metathesis (SMM) [51], a novel approach that expands the utility of sortase beyond traditional ligation to a metathesis reaction compatible with full-length endogenous proteins. This technique uses an engineered calcium-independent sortase variant called 6M-Srt, because calcium levels in the nucleus are likely too low to support sortase activity. To enhance its catalytic efficiency and improve affinity for the positively charged histone H3, the 6M-Srt was engineered by removing positively charged residues and introducing negatively charged ones near the substrate binding pocket. The method employs synthetic histone peptides fused to cell-penetrating peptides (CPPs) to facilitate intracellular delivery, and demonstrated that they could introduce PTMs such as biotinylation, dimethylation, and crotonylation on histone H3 on reconstituted nucleosomes, in isolated nuclei, and in live cells. In addition, they observed a modest reduction in GFP reporter expression following the introduction of repressive marks such as H3K9me3K27me3; however, because the system lacked locus-specific targeting and did not include direct measurements of chromatin state at the GFP locus, the causal link between histone editing and transcriptional repression remains suggestive rather than definitive. While the editing efficiency in live cells was approximately 15% and non-specific labeling of other proteins containing sortase recognition motifs was observed, this study represents a step forward in applying sortase-mediated histone modification within living cells. Pelaz et al. developed a high-throughput platform for screening chromatin interactions using pre-assembled tailless nucleosomes immobilized on 96-well plates in which histone tails can then be installed by SML (Figure 2C) [52]. This streamlined approach enabled efficient (>80%) ligation using LPATG-depsipeptides, though it uses the non-native sequence compared with the natural APATG motif. In this approach, the nucleosomes have a biotin tag on their DNA and are immobilized on streptavidin-coated wells. Therefore, unreacted components from the ligation reaction can simply be washed away. Using this system, the authors screened interactions between eCFP-tagged human HP1β and modified nucleosomes, and found that HP1β, a reader of H3K9me2, bound more strongly to nucleosomes bearing both H3K9me2 and H3S28ph than to those with H3K9me2 alone.

##### Other methods of nucleosome editing

Other *in cellulo* and *in vivo* histone and nucleosome editing techniques are under development: one example is a split-intein strategy allowing researchers to attach N-terminal peptides onto H3 inside live mice, although it currently only modifies a small, AAV-transduced neuron population for a brief period [53]. These early stage advances signal that precise *in cellulo* and *in vivo* histone editing could be the pivotal next step for untangling PTM cross-talk on chromatin.

Beyond chemical and semisynthetic approaches, genetic code expansion to introduce modified Lys residues has been elegantly applied to generating histones [54]. Acetyl-Lys and methyl-Lys residues have been installed site-specifically into histones using unnatural amino acid mutagenesis using a nonsense suppression strategy [55,56]. One limitation of this method is that incorporating two or more PTMs into one histone is generally challenging due to decreased expression efficiency [57].

#### Asymmetric nucleosomes

Native nucleosomes contain two copies of each core histone, but it’s unclear how frequently each histone copy in a nucleosome has identical PTMs *in vivo*. To study PTM cross-talk *in cis* (PTMs on the same histone molecule) versus *in trans* (PTMs on different copies of the same histone), asymmetric nucleosome systems have been developed to incorporate distinct histone modifications, mutations, or variants on each half of the octamer. These engineered nucleosomes have become useful tools for dissecting the functional consequences of chromatin modification patterns. Early methods of asymmetric nucleosome preparation utilize two pools of histone H3 with either orthogonal affinity tags or both tagged and untagged H3. In the former case, sequential affinity purification yields tetramers with one copy of each tagged H3, which are mixed with H2A/H2B dimer for nucleosome reconstitution [58]. In the latter case, a large excess of untagged H3 is used in octamer refolding, resulting in primarily symmetric, untagged octamers, and a small proportion of asymmetric, singly tagged octamers that could be affinity purified [59]. Subsequent approaches employed enzymatically cleavable tags for crosslinking two differently modified pools of histone H3. Crosslinking is achieved either by DTNB-directed asymmetric disulfide formation between two cysteine residues within the tags on H3 [60], or by tagging the two pools of H3 with SpyCatcher and SpyTag [61]. Recently, SML was used to generate asymmetric nucleosomes. This approach employed an engineered thermostable sortase with a circularized backbone, cW11, which operates under mild conditions and exhibits ligation efficiencies of 80–90% on histone H3 in the context of tailless nucleosomes [49]. Stepwise SML can be performed with the use of anion exchange chromatography to separate nucleosomes by the number of ligated H3 tails (0, 1, or 2), permitting the production of nucleosomes with differently modified H3 tails (Figure 2B). These nucleosomes were used to probe PTM cross-talk *in cis* (on the same tail) and *in trans* (on different tails).

#### Nucleosome assembly techniques

Purification of histone octamers for *in vitro* nucleosome assembly traditionally is challenging, relying on individual expression of core histones in *E. coli*, purification under denaturing conditions, *in vitro* reconstitution into octamers, and salt gradient dialysis into nucleosomes. These steps can be labor-intensive and prone to variability. Improvement in these techniques can facilitate *in vitro* nucleosome production for applications in studying PTM cross-talk. For example, Zhao et al. highlighted the importance of precise octamer-to-DNA ratios in achieving efficient nucleosome assembly and introduced a kinetic model to help understand phases of nucleosome assembly through FRET and fluorescence thermal shift (FTS) assays [62]; Shim et al. introduced a single-plasmid polycistronic system that enables non-denaturing co-expression and purification of histone octamers, eliminating the need for refolding [63].

Advances in mononucleosome synthesis have provided insight into many specific chromatin mechanisms; however, higher-order nucleosome arrays are required to interrogate chromatin function in contexts where inter-nucleosomal interactions, spacing, and geometry are critical [64,65]. Nucleosome arrays can be generated through several complementary approaches, including cell extract–based assembly, remodeler-driven assembly, and the widely used linear salt gradient dialysis [66]. These core strategies can be combined with cloning-based DNA assembly, modular or ligated nucleosome construction, and chemical post-assembly modification to achieve precise control over array composition and architecture [67]. In many cases, DNA templates consist of tandem Widom 601 positioning sequences separated by defined linker DNA, with optional incorporation of additional binding motifs to recruit transcription factors, chromatin remodelers, or sequence-specific targeting domains [68].

Early work by Bulger et al. demonstrated that regularly spaced nucleosome arrays can be assembled *in vitro* using DNA, purified core histones, ATP, and *Drosophila* chromatin assembly factors, dCAF-1 and dCAF-4, without reliance on salt dialysis; these factors, fractionated from embryo extracts, were sufficient to rapidly generate periodic arrays with repeat lengths resembling native chromatin [69]. Krietenstein et al. showed that remodeler activity alone is sufficient to establish nucleosome-free regions and phased arrays with remodeler-specific spacing across genomes on reconstituted chromatin [70]. Arrays assembled by salt dialysis can be further organized by ATP-dependent remodelers: Oberbeckmann et al. reconstituted nucleosome arrays on genomic DNA at defined densities and aligned them against physical barriers, then applied purified remodelers and MNase-seq to show that remodeler identity determines nucleosome spacing and phasing through intrinsic ruler elements, largely independent of DNA sequence or nucleosome density [71].

DNA-barcoded nucleosomes provide a high-throughput platform for retrieving unique modification profiles of individuals inside a nucleosome library. Dann et al. exploited this by adding a unique hexanucleotide barcode to one end of the Widom 601 DNA sequence for each nucleosome bearing a different histone PTM, then pooling over 100 species for a single “one-pot” remodeler assay [72]. A restriction site embedded inside the DNA is initially buried (“blocked”) within the wrapped nucleosome; then, ISWI (Imitation SWItch) remodeler complexes expose the site (“unblocked”), which gets cleaved immediately. The disappearance of the intact barcoded fragment quantitatively tracks ISWI-driven nucleosome repositioning. This barcode-coupled restriction-enzyme assay allows multiplex screening that maps remodeler specificity and substrate preference, as well as kinetics across hundreds of uniquely modified nucleosomes.

More chemically defined approaches have enabled precise manipulation of array composition. Ge et al. assembled designer 12-mer nucleosome arrays from tetranucleosome building blocks containing defined combinations of unmodified, H3K27me3-modified, or mutant histones using Widom 601 repeats and tandem DNA ligation, revealing that PRC2-mediated H3K27 methylation propagates preferentially to neighboring nucleosomes *in cis* and is constrained by array geometry unless overridden by the regulatory subunit JARID2 [73]. Complementary advances in DNA construction were reported by Spakman et al., who generated nucleosome arrays with precisely defined linker length and sequence using an iterative Gibson Assembly strategy followed by salt dialysis; these substrates enabled single-molecule force spectroscopy experiments demonstrating stepwise nucleosome unwrapping with partial histone retention at high tension [74].

Blacketer et al. developed a ligation-based strategy to assemble ordered di-, tri-, and tetranucleosome arrays by sequentially ligating mononucleosomes reconstituted on Widom 601 positioning sequences joined by unique non-palindromic restriction sites [67]. Using sedimentation and differential centrifugation assays, they showed that tetranucleosome arrays undergo limited intra-array compaction but robust inter-array self-association, and that histone H4 tails play a dominant role in mediating inter-array interactions while having minimal impact on intra-array compaction in short arrays.

The variety of techniques used to make nucleosomes and nucleosome arrays has enabled the field to take the first steps in probing chromatin structure and function. Used in combination with technical advances in mass spectrometry, omics technology, and synthetic chromatin interactors, these advances have helped uncover intricate epigenetic mechanisms.

#### Mass spectrometry-based analysis

Initial mass spectrometry (MS) on histones used bottom-up proteomics approaches to identify individual histone PTMs. This method employs trypsin to generate short histone tail peptide fragments (typically 5–10 amino acids), enabling identification of individual PTMs but generally lacking the resolution to detect combinatorial modifications. More recently, middle-down MS enabled analysis of larger histone tail fragments (30–50 amino acids), preserving PTM combinations. It does so by using proteases that cleave selectively to yield longer peptides.

Bottom-up MS can be combined with a middle-down approach. Janssen et al. developed an integrated workflow in which both tryptic bottom-up peptides and GluC-generated middle-down peptides are both analyzed in a single LC-MS run [75]. This allows quantification of single-site PTMs with high sensitivity, from the tryptic peptides, while simultaneously profiling combinatorial cross-talk patterns on intact H3/H4 N-terminal tails, bridging bottom-up and middle-down analyses. Because the workflow relies on a single formic-acid buffer set and one LC gradient, any standard proteomics lab can seamlessly adopt it to perform streamlined histone cross-talk MS analyses.

**Figure 3:**
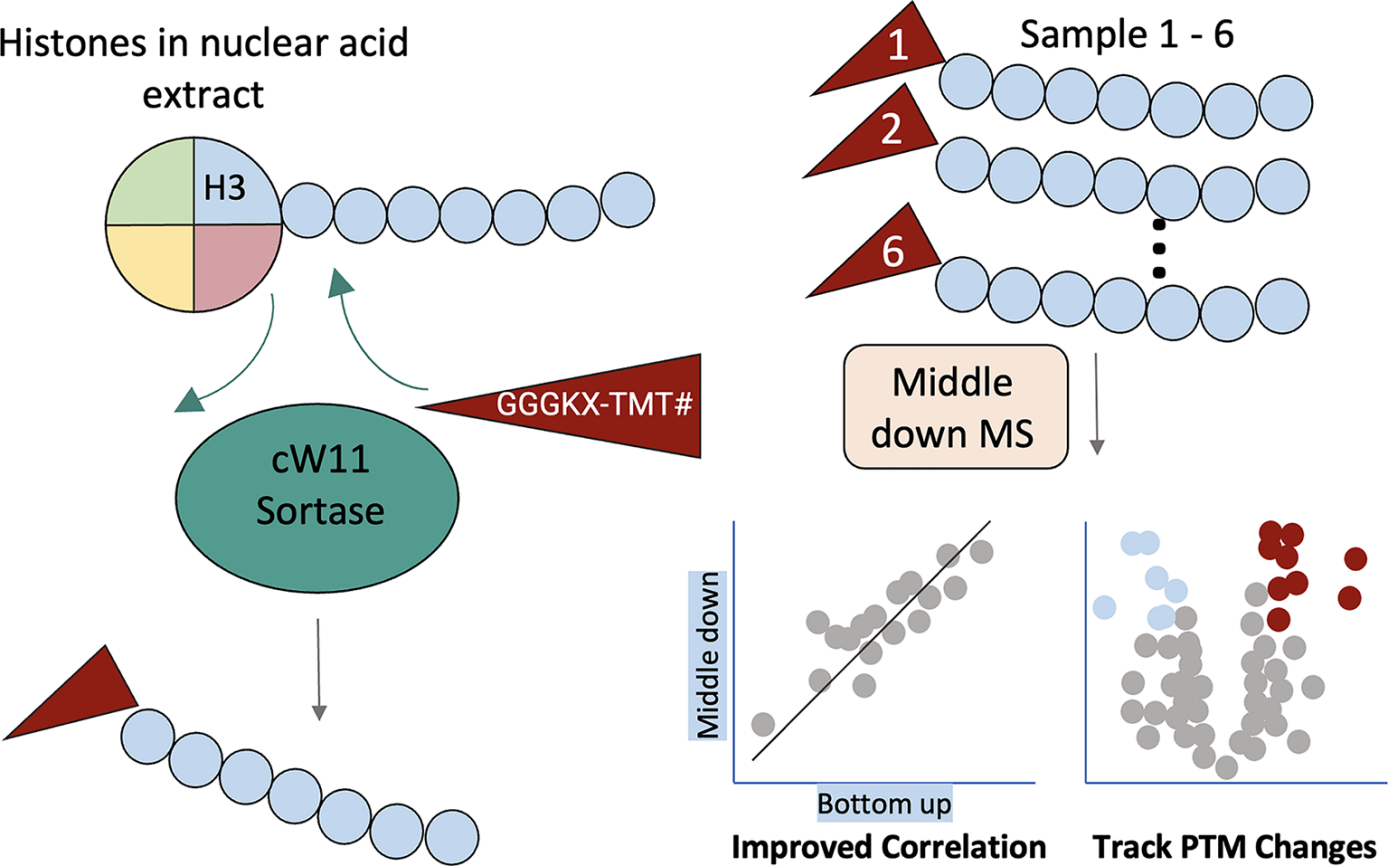
“Cut and Paste”: cW11 sortase-mediated TMT tagging of histone H3 tails enables multiplexed middle-down analysis of PTM cross-talk Histones are isolated from nuclear acid extract and reacted with cW11 sortase and an N-terminal GGGKX-TMT peptide to append TMT tags specifically to H3 tails. Labelled histone H3 tails from multiple samples (1–6) are then combined and analyzed by middle-down mass spectrometry. Quantitative middle-down measurements show improved agreement with conventional bottom-up proteomics and allow tracking of condition-dependent changes in histone PTM patterns across samples.

To enable multiplexed middle-down histone proteomics, a variant on SML was developed to cleave the histone H3 tail and append an oligoglycine peptide pre-functionalized with a tandem mass tag (TMT) (Figure 3) [49]. This one-pot “Cut-and-Paste” can be performed in crude nuclear extracts, replacing the traditional histone purification, GluC digest, and weak-cation-exchange hydrophilic-interaction (WCX-HILIC) chromatography optimal for GluC histone peptides. TMT multiplexing enabled quantification of ∼150 distinct H3 proteoforms and their changes in response to drug treatment.

#### Omics technology/chromatin profiling technologies

While *in vitro* histone cross-talk studies provide clarity in mechanistic insights, *in cellulo* studies allow understanding of histone cross-talk regulation with genomic coordinates. Common chromatin profiling approaches (e.g., ChIP-seq, CUT&RUN, CUT&Tag, and ATAC-seq) can provide a powerful “snapshot” of the heterogeneous chromatin state, and usually a single PTM is assayed per experiment. Current limitations lie within obtaining single-cell measurements, measuring multiple PTMs in the same cells, and measuring the temporal ordering of chromatin events. Recent technological advances are beginning to address these gaps by enabling the generation of higher-resolution, multi-mark, single-cell and time-resolved data that bring us closer to measuring true cross-talk dynamics on the same nucleosome and across transcriptional states.

Co-ChIP (combinatorial indexed ChIP) was designed to quantify co-occurrence of histone marks on single nucleosome/chromatin fragments, rather than merely overlapping the signal from two separate ChIP-seq experiments that target different PTMs [76]. It uses sequential immunoprecipitation (IP) with barcoding. First, chromatin is pulled down with beads that have an antibody to PTM “A” and ligated to indexed DNA adapters that uniquely label individual chromatin fragments. Next, the labeled material is released, pooled, and subjected to a second IP for PTM “B,” and another unique DNA barcode is added to label fragments that carry PTM B within the PTM A-tagged pool. Sequencing reads would then allow specific (A, B) pairs to be identified that co-occur on the same nucleosome or chromatin fragment. Compared with merely sequential IP, this method is highly scalable and multiplexable.

Similar to ChIP, conventional CUT&Tag (and CUT&RUN) assays typically profile one epitope per reaction, so multi-epitope interpretation often relies on comparing or overlaying tracks generated from separate aliquots of cells, which cannot directly distinguish true co-occurrence of PTMs on the same nucleosome. To address this, Multi-CUT&Tag was developed as an adaptation of CUT&Tag in which protein A-Tn5 transposase fusion proteins are bound to individual antibodies and unique DNA barcodes (“adapters”), and the resulting antibody-protein A-Tn5 conjugates are applied to the same cells [77]. Distinct antibody-pA-Tn5 complexes target different histone marks, make a cut in the genome near the bound site, and insert their barcoded adapters. “Mixed-barcode” fragments can arise when two antibody-pA-Tn5 complexes act in proximity, providing evidence for local co-occurrence of PTMs.

Like standard CUT&Tag, the approach leverages enzyme-tethered generation of small chromatin fragments around the bound target, which improves specificity and signal-to-noise relative to ChIP-seq, which uses bulk-fragmentation. Important caveats are that only two antibody barcodes can appear on a single genomic insert, and as the number of conjugates increases, co-localizing complexes may compete for insertion into the same DNA fragments; moreover, co-association readouts may still be influenced by steric constraints, and mixed reads do not always resolve whether two marks are on the same nucleosome versus adjacent nucleosomes.

Hi-Plex CUT&Tag was developed to improve the scalability of multiplex CUT&Tag [78]. In this approach, antibodies are first biotinylated and converted into “barcoded antibody” reagents by coupling them to streptavidin loaded with adaptor oligos that contain antibody-specific barcodes and Tn5 recognition sequences. Next, barcoded antibodies are pooled and incubated with the same permeabilized cells. After washes remove unbound antibodies, Tn5 (unloaded, without DNA) is added to generate sequencing fragments, enabling highly multiplexed profiling (demonstrated with 36 mAbs plus an IgG control in one sample) while reducing barcode cross-contamination by physically attaching barcodes to antibodies rather than to Tn5. Its core co-localization signal (“heterotone” fragments carrying two different barcodes) is inherently distance-bounded, requiring two proximal events within ∼1 kb and using fragment length as a proxy for separation, so it does not guarantee single-nucleosome (∼147 bp) resolution for mapping PTM cross-talk occurring on the same nucleosome but could be useful for mapping more distant events.

At the single-cell level, approaches such as CoBATCH and MAbID expand the chromatin-profiling toolkit by enabling scalable, high-throughput measurements that preserve cellular heterogeneity. Single-cell resolution is an important advantage for studying histone cross-talk, since epigenetic modifications are often lineage-restricted or transient along differentiation trajectories and can be obscured in bulk averages [79,80].

ChOR-seq and SCAR-seq are replication-coupled ChIP-seq methods [81] that allow sequencing of nascent DNA that was associated with PTM of interest, by labeling newly replicated DNA with EdU (5-ethynyl-2′-deoxyuridine) and then perform ChIP on them. ChOR-seq allows quantification of the time course of PTM re-establishment on nascent DNA after fork passage. SCAR-seq adds strand separation to compare PTM occupancy on nascent sister chromatids. These methods give us time-resolved information on PTM establishment after fork passage, and could be very useful to apply to PTM cross-talk studies.

Taken together, *in cellulo* measurement is moving from inferring “cross-talk” by overlaying separate single-mark maps to direct measurement of combinatorial chromatin states, as well as developing new techniques that have single-cell resolution and temporal resolution. This shift is essential for making cross-talk models predictive across cell states and tissues. These tools complement *in vitro* cross-talk studies by anchoring PTM relationships to genomic coordinates, but an important limitation remains: most measurements are still snapshots of co-occurrence or co-localization, and do not by themselves resolve temporal ordering or causality.

#### PTM probes

A growing number of PTM probes are enabling the precise investigation of histone cross-talk in both live cells and reconstituted nucleosomes. These tools help visualize or detect combinations of histone marks and uncover how PTMs influence each other *in cis* or *in trans*. Peptide-based studies have revealed many PTM cross-talk and PTM–reader interactions, but they often miss how other parts of the nucleosome shape these interactions. Ruthenburg et al. addressed this by demonstrating that the BPTF tandem PHD finger and bromodomain cooperatively recognize H3K4me3 and H4K16ac on the same nucleosome, and that binding is significantly more selective at the possible acetyl-lysine sites in the nucleosome context compared with isolated peptides [82]. The coexistence of these modifications within single nucleosomes in human cells was confirmed with ChIP and demonstrated genomic co-localization of BPTF with these marks. Similarly, using the tandem PHD-bromodomain module of BPTF as a reader, Marunde et al. showed that its recognition of dual H3 marks—H3K4me3 together with H3K14ac or H3K18ac—is significantly enhanced in the nucleosome context compared with peptides [83]. This work demonstrates that reader affinity and specificity are tightly modulated by nucleosome structure, and highlights the utility of engineered readers as biochemical multivalent tools to probe histone PTM cross-talk.

Reader proteins that can sense two neighboring PTMs at once can be used to decipher histone-mark cross-talk, yet systematic tools for that task have been scarce. Chen et al. have investigated tandem reader domains against a combinatorial H3 peptide library [84]. Their screen revealed that BPTF binds H3K4me3 far better when K14 is acetylated, but loses affinity when K9 is acylated and completely loses binding by T3 phosphorylation.

**Figure 4:**
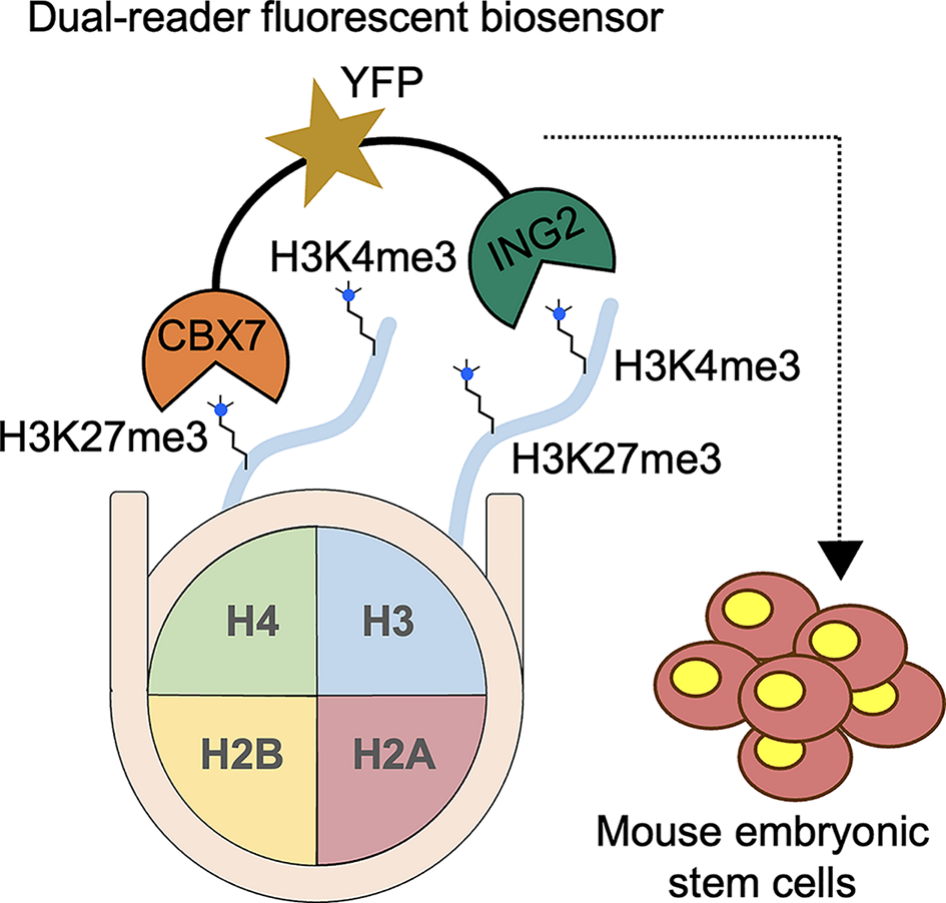
Dual‐reader fluorescent biosensor (“cMAP”) selectively reports bivalent H3K4me3/H3K27me3 nucleosomes in living mESCs A nucleosome is shown with its four core histones (H2A, H2B, H3, H4) color-coded in cross-section. Two H3 N-terminal tails (purple) bear H3K4me3 and H3K27me3, on the same tail. The biosensor is a modular fusion of the ING2 PHD finger (green; selective for H3K4me3) and the CBX7 chromodomain (tan; selective for H3K27me3) linked by a flexible peptide spacer that carries a yellow fluorescent protein Venus (YFP, star) reporter. Engagement of both reader domains with their targets stabilizes binding only when the two PTMs occur on the same nucleosome, producing bright nuclear puncta (right) in mouse embryonic stem-cell (mESC) nuclei. This design enables live-cell visualization of bivalent chromatin domains and interrogation of PTM cross-talk.

Delachat et al. show that individual reader domains can be stitched together into a multivalent biosensor [85]. Their probe, cMAP3, fuses the H3K27me3-recognizing CBX7 chromodomain to the H3K4me3-recognizing ING2 PHD finger through an optimized nine-residue neutral and flexible linker and is expressed as a GFP fusion. Microscale thermophoresis assay revealed a *K*_d_ of 170 nM for a peptide bearing both marks, more than ten-fold tighter than for peptides containing either modification alone (Figure 4). When transiently transfected into mESCs, cMAP3 forms bright nuclear foci, but these puncta disappear if either reader domain is point-mutated. The sensor also reports chromatin dynamics: 24 h treatment with the PRC2 inhibitor UNC1999, which inhibits H3K27 methylation, disperses the foci, whereas HDAC or LSD1 inhibition leaves them unchanged; washing out UNC1999 allows the clusters to re-form. Thus, cMAP3 provides a genetically encoded, reversible readout of bivalent histone PTMs.

Other histone PTM multivalent readers include but are not limited to UHRF1 (tandem Tudor domain and PHD domain recognizing H3K9me3 and unmodified H3R2) [86], BRD4 (tandem bromodomains recognizing acetylated lysines on H3 and H4) [87], TAF1 (tandem bromodomains recognizing acetylated H4 at K5, K8, K12) [88], TRIM24 (PHD finger and bromodomain, recognizing unmodified H3K4, trimethylated H3K9, acetylated H3K18 and acetylated H3K23) [89,90], TRIM33 (PHD finger and bromodomain recognizing unmodified H3K4, trimethylated H3K9 and acetylated H3K18) [89], CHD4 (tandem PHD fingers) [91], ZMYND8 (PHD finger and bromodomain recognizing H3K4me1 and H3K14ac) [92], and ZMYND11 (bromodomain and PWWP domain recognizing unmodified H3.3S31 and trimethylated H3.3K36) [93,94]. These multivalent proteins present valuable opportunities to (1) explore PTM cross-talk in their native context and (2) leverage their modular architecture to engineer custom readers with multivalent reader domains capable of recognizing specific PTMs on demand.

### Examples of histone PTM cross-talk

In the early 2000s, studies describing instances of histone cross-talk began to emerge [95,96]. Today, more than 200 histone residues are known to be modifiable, and at least 75 distinct PTMs have been documented [97,98]. These marks frequently act in concert, with one modification altering the writing, erasing, or reading of another. There have been many attempts to understand the biological significance of such cross-talk.

#### Methyltransferase

##### Dot1/DOT1L

The Dot1(yeast)/DOT1L(human) methyltransferases are associated with active transcription through interactions with the SET1/mixed lineage leukemia (MLL) complexes and super elongation complex [97]. Beyond transcriptional control, DOT1L also supports genome stability and DNA damage responses, is essential for development and hematopoiesis, and is a key dependency in MLL-rearranged leukemias [99]. Dot1/DOT1L methyltransferase activity modifies H3K79, with the most pronounced activity at transcription start sites (TSS), and diminishing activity through the gene body [100]. This reflects both the reversible association of Dot1/DOT1L with the super elongation complex, and its stimulation by histone PTMs enriched at TSS. Shahbazian et al. as well as Nakanishi et al. found that Dot1/DOT1L methylation is additively enhanced by multiple histone modifications concentrated at active TSS, including acylation of H4K16 and H3K9, and most notably ubiquitination of H2BK120 [96,101,102]. The influence of H2BK120 ubiquitination on Dot1-catalyzed methylation of H3K79 was one of the early examples of cross-talk validated enzymologically and genetically. The stimulatory effects of H2BK120Ub and H4K16ac both arise from stabilization of the catalytically competent configuration of DOT1L and H3K79, leading to an increase in *k*_cat_ [103,104]. In the absence of histone modifications, DOT1L engages the nucleosome using a flexible loop to contact the acidic patch between histones H2A and H2B. Ubiquitination of H2BK120 acts as an additional anchor for DOT1L by constraining its mobility and pushing it closer to a catalytic pose through hydrophobic interactions between the C-terminus of ubiquitin and the DOT1L αK helix [105,106]. The orienting effect of H2BK120Ub on DOT1L yields a 6- to 10-fold increase in *k*_cat_, without significantly altering DOT1L *K*_m_ or *K*_d_.

Nonetheless, Worden and colleagues found that methylation of H3K79 requires reorganization of the nearby H3 segment aa77-81, which in turn requires participation of histone H4 R17 through R19 [107]. In the presence of H2BK120Ub, the sequence surrounding H3K79 and H4 R17-R19 adopts two distinct conformations, only one of which is catalytically active [107]. In addition, Valencia-Sanchez and colleagues found that in the presence of H4K16ac, these two populations coalesce into a single catalytically active conformation mediated by hydrophobic and hydrogen bond interactions between H4 A15 to R17 and Dot1 [104]. The positioning effect of H4K16ac enhances Dot1 catalysis 6 to 18-fold in isolation, and cooperates with H2BK120Ub to enhance *k*_cat_ ∼7- to 35-fold [104].

Cross-talk between Dot1/DOT1L and H3K9 acylation is mediated by super elongation complex components AF9 and ENL, which bind acyl-modified lysines through their YEATS domains [102]. The YEATS domain exhibits preferential affinity for medium chain, unsaturated acyl modifications (crotonyl > methacrylyl > butyryl > acetyl), linking Dot1/DOT1L activity to metabolism and the microbiome [108,109]. The Dot1/DOT1L binding helices of AF9 and ENL are frequently found fused to MLL in various leukemias, which has motivated the development of DOT1L inhibitors as potential chemotherapeutics.

##### MLL

The SET1 and MLL family of methyltransferases install H3K4 methylation, which accompanies gene activation in organisms from yeast to humans. These enzymes can orchestrate gene programs in development, hematopoiesis, and stem-cell self-renewal, and their rearrangements drive acute leukemias—making the pathway a key therapeutic target [110]. The methyltransferase activity of SET1/MLL enzymes is most pronounced at TSS and diminishes with progress into the gene body [111,112]. Catalytic activity is linked to both ubiquitination of histone H2BK120 and acetylation of histone H3, both of which co-localize at active TSS [96,101].

Cross-talk with H2BK120 ubiquitination has the greatest stimulatory effect on H3K4 methylation by MLL, increasing both the rate of methylation, and favoring trimethylation. This is attributed to the orienting effects of ubiquitin, which interacts with Retinoblastoma-binding protein 5 (RBBP5) in humans or SWD1 in yeast through the ubiquitin Ile44 hydrophobic patch, and orders the Set1 RxxxRR helix, stabilizing an interaction between the helix and the H2A/H2B acidic patch [113]. The effect of acetylation has been ascribed to increased histone tail mobility, with the strongest stimulatory effects from H3K18ac and H3K23ac [114,115].

##### PRC2

The polycomb methyltransferase complex 2 (PRC2) regulates epigenetic silencing through H3K27 methylation, the defining feature of facultative (reversible) heterochromatin. Its role in heterochromatin formation contributes to transcriptional repression in development, cell-fate decisions, and X-chromosome inactivation [116]. Dysregulation of PRC2 methyltransferase activity is a driver of numerous cancers, which has spurred the development of several potent inhibitors, including the FDA-approved tazemetostat [117]. PRC2 comprises the SET domain catalytic subunit Enhancer of Zeste Homolog (EZH1 or EZH2), SUZ12, Embryonic ectoderm development protein (EED), and RBBP4 or RBBP7 [118]. PRC2 methyltransferase activity is modulated by multiple lysine methylation-dependent interactions with both histone and non-histone proteins [119]. Subunits EED and EZH2 discern regions of active transcription through H3K4me3 and H3K36me3, both of which inhibit complex activity, preserving the activated state [120,121]. Conversely, subunits EED, Jumonji, AT-rich interaction domain 2 (JARID2), and Adipocyte enhancer-binding protein 2 (AEBP2) sense transcription repression through H3K27me3 and H2AK119Ub, and respond by enhancing methyltransferase activity, entrenching the repressive chromatin environment [64].

**Figure 5:**
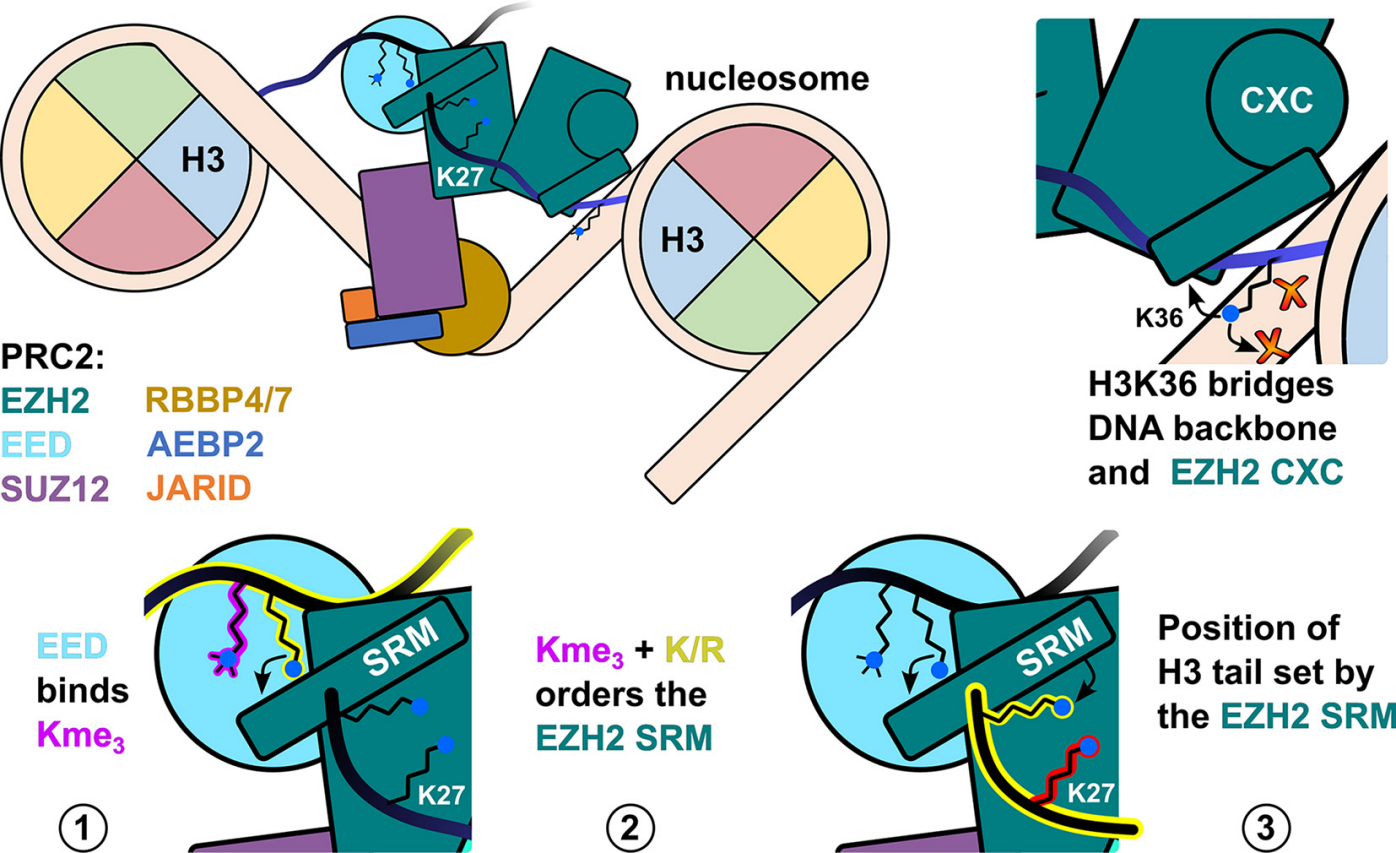
PRC2 cross-talk with H3 modifications in the mono- and di-nucleosome context PRC2 methyltransferase EZH2 undergoes allosteric activation when subunit EED binds to H3K27me2/3 on an adjacent nucleosome (bottom left). This binding orders and shifts the EZH2 SRM helix, which stabilizes binding to the EZH2 substrate peptide (bottom right). H3K36 on the nucleosome substrate of EZH2 bridges between the DNA phosphate backbone and a helix of the CXC domain (top right). The bridging effect of H3K36 is disrupted by trimethylation, which in turn inhibits EZH2 methylation of H3K27 on the H3K36me3 modified tail.

The PRC2 subunit EED scaffolds the methyltransferase EZH2 and selectively binds to trimethylated lysine residues (Figure 5). The local peptide sequence of the trimethylation site determines whether the binding stimulates or inhibits EZH2 activity. Ge et al. determined that when the residue flanking the trimethylation site is a lysine or arginine (e.g. H3R26), it repositions the stimulus-response motif (SRM) helix of EZH2, increasing *k*_cat_ 5- to 8-fold [73]. Conversely, no repositioning of the SRM is observed when H3K4me3 binds due to adjacent residues H3T3 and H3Q5, decreasing *k*_cat_ 3- to 5-fold [73]. Moreover, H3K4me3 competes with H3K27me3 and the similarly SRM-repositioning complex subunits LCOR K1241me3 (PRC2.1) and JARID2 K116me3 (PRC2.2), compounding the inhibitory effect of H3K4me3 on EZH2 [119,122]. Notably, Lee and colleagues found that PRC2 complexes containing methyltransferase EZH1 are not strongly stimulated by SRM-dependent mechanisms due to divergence in SRM sequence of EZH1 [123]. Unmodified H3K4 peptide has also been reported to bind RBBP4/RBBP7; however, this has no effect on nucleosome binding and the same binding pocket on RBBP4/RBBP7 is typically occupied by subunits AEBP2 and Suz12 [124,125].

The proximity of H3K36me2/3 to the nucleosome DNA defines its unique mode of PRC2 inhibition. Finogenova et al. found that in the unmethylated state, H3K36 interacts closely with the DNA phosphate backbone, positioning PRC2 on the nucleosome through the EZH2 bridge helix and CXC domain [121]. Di- and trimethylation of H3K36 prevents this close interaction with the DNA phosphate backbone, which propagates through the bridge helix and CXC domain to the catalytic domain, thereby decreasing *k*_cat_ 5-fold without significantly changing the nucleosome *K*_d_ [122,126]. This inhibitory effect of H3K36me2/3 can be mitigated by making mutations at the interface between EZH2 CXC and catalytic domains (e.g. M700A, M700V, K634E) [126].

More recently it has been suggested that PRC2 activity is restricted by ubiquitination of H3 by the ubiquitin ligase Ubiquitin-like, containing PHD and RING finger domains, 1 (UHRF1) [127]. UHRF1 ubiquitination targets include H3K14, K18, K23, K27, and K36 [128], any of which could plausibly disrupt PRC2 binding and catalysis. Current structures of PRC2 bound to the H3 tail fail to resolve histone residues prior to Q19 due to the tail’s flexibility, however these structures show H3Q19 within a few angstroms of the SRM helix. When the SRM helix is in the catalytically favorable configuration (i.e. the position induced by JARID2 K116me3 binding), it moves within hydrogen bond distance of the H3Q19 side-chain [64,122]. It is thus tempting to speculate that a bulky ubiquitination at H3K18 or K14 is likely to disrupt any substrate-positioning effect of the SRM helix.

##### SETDB1

**Figure 6:**
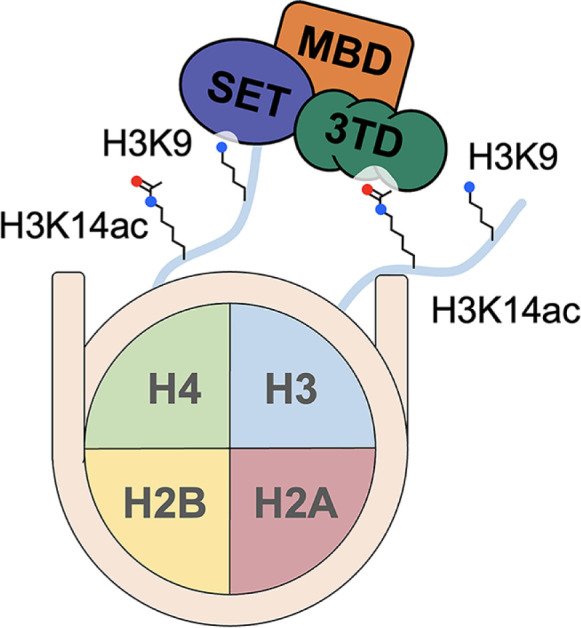
Model of the interaction between nucleosome and the SETDB1 complex for H3K9 methylation The triple Tudor domains (3TD) interact with H3K14ac and recruit the SETDB1 complex to the nucleosome. These interactions promote the methylation of H3K9 at the *trans* position.

The SET domain bifurcated histone Lys methyltransferase 1 (SETDB1) is one of the major enzymes that induce H3 Lys9 (H3K9) methylation [129]. SETDB1 is linked to tumorigenesis through H3K9 methylation-induced gene silencing, inhibition of cell cycle arrest, and stimulation of cell proliferation [130]. SETDB1 contains triple Tudor domains (3TD; TD1, TD2, and TD3) that act as binding domains for methylated Lys and Arg, a methyl-CpG binding domain (MBD), and a SET domain with a catalytic active site for methylation [129]. The SET domain is split into the Pre-SET, Bifurcated SET, and Post-SET domains, all of which are associated with the recognition and methylation of Lys (Figure 6) [129,131].

It was recently identified that SETDB1 co-localizes with chromatin that appears to have H3K9 methylation (H3K9me) and H3K14ac acetylation (H3K14ac). *In cellulo* studies in combination with ChIP-Seq observed the co-localization of H3K14ac on SETDB1-associated K9me3 peaks [132]. Additionally, a knockout cell line for HBO1, an acetyltransferase responsible for H3K14 acetylation, showed a significantly reduced level of H3K14ac and H3K9me3 [132]. Knockdown of H3K14/H3K9 acetyltransferase Gcn5 in Drosophila embryo by Tang and colleagues reduced the deposition of H3K9me3 and accumulation of GFP-SETDB1 on nuclear foci [133]. These results suggest the involvement of H3K14ac in the SETDB1-mediated K9 methylation [129,132].

Binding studies by Jurkowska and colleagues identified that the triple Tudor domains in SETDB1 specifically target the doubly modified H3K9me/K14ac tails (Figure 6). Modified H3 (4-19) peptides that have mono-, di-, or tri-methylated at K9 and acetylated at K14 marks showed their low micromolar range of *K*_d_ with triple Tudor domains. While singly modified H3K14ac shows weak (millimolar) binding to the triple Tudor domains, neither H3K9me3 nor unmodified peptides shows binding [129]. Among the three peptides (i.e., H3K9me/H3K14ac, H3K9me2/H3K14ac, and H3K9me3/H3K14ac), the H3K9me2/H3K14ac peptide exhibited a higher binding preference for the triple Tudor domains [129]. The crystal structures obtained from X-ray diffraction reveal that the specificity of the H3K9me2/H3K14ac peptide arises from its rigid fit within TD2 and TD3, and the linker region between them, through hydrogen bonding and hydrophobic interactions [129]. Each Tudor domain revealed a distinct role in the interactions with H3K9 methylated peptide: TD1 weakly affects peptide binding, TD2 is mainly involved in the interactions with H3K9me3, and TD3 prefers the binding with H3K9me1/2 [129].

The influence of H3K14ac on H3K9 methylation by SETDB1 depends on the position of H3K9, as there are two histone H3 proteins in a nucleosome (Figure 6) [132,134]. Experiments by Chandrasekaran et al. with modified peptides and nucleosomes suggest the recruitment of SETDB1 to nucleosomes is by H3K14ac interacting with the triple Tudor domains. The binding of H3K14ac to the triple Tudor domains prevents the recognition of K9 on the same histone tail by the SET domain, where the catalytic center is located, thereby inhibiting its methylation. However, this interaction may help the recruitment of SETDB1 to the nucleosome and induce the position of the SET domain towards the other tail, promoting the methylation of H3K9 at the trans position [132]. In addition to cross-talk between H3K14ac and H3K9me, SETDB1 also suggests its role in other epigenetic repressive modifications. In a SETDB1 KO cell line, a reduction in H3K9me3 signal and DNA methylation was observed [132]. *In cellulo* studies with primordial germ cells (PGCs) indicated that knockout of SETDB1 induced a decrease of not only H3K9me3 but also H3K27me3 and DNA methylation [135]. Further elucidation is needed to understand the underlying mechanisms of these aspects.

#### Demethylases

Histone demethylases play a vital role in cellular homeostasis, development, and disease progression by fine-tuning gene expression through the removal of methyl groups from mono-, di-, or tri-methylated Lys residues (Kme1/2/3). Given the regulatory impact conferred by these methyl marks, demethylase regulation is often linked to key cellular processes - including inflammation and antiviral signaling (e.g., KDM4D demethylase regulating the TLR4/TIRAP/MyD88/NF-κB axis; KDM5A demethylase modulating PI3K/AKT/S6K1/PD-L1 signaling; KDM5B/C impacting STING expression [136]), as well as cellular state transitions like epithelial- or endothelial-to-mesenchymal transition (LSD1 demethylase [137]), and cell cycle progression (PHF8 demethylase [138]).

##### JMJD and LSD1/2

All known classes of histone demethylases require molecular oxygen and generate formaldehyde as a byproduct. Among them, the Jumonji C domain-containing demethylases (JMJDs) utilize α-ketoglutarate and an iron cofactor to catalyze the removal of methyl groups via radical-mediated oxidation [139]. In contrast, the flavin-dependent Lys-specific histone demethylases (LSD1 and LSD2) employ a flavin adenine dinucleotide (FAD) cofactor to demethylate mono- or di-methylated H3K4 via a direct hydride transfer mechanism from the methyl group to FAD [140–142].

Following demethylation, histone tails lose chemical groups that regulate the binding affinity and dwell time of chromatin regulators, which recognize distinct methyl-lysine states through reader domains, ultimately altering downstream chromatin remodeling and gene regulation. While lysine methylation doesn't affect the Lys residue’s permanent charge, it affects demethylases’ target selectivity. For example, trimethylation fixes the ε-amine’s positive charge, preventing the formation of a neutral Lys that LSD1/LSD2 require for catalysis [143,144]. This biochemical constraint explains substrate selectivity among demethylases and affects which methylation states are accessible to specific chromatin readers. A recent nucleosome structure suggests that unmethylated Lys4 on histone H3 fits snugly into the minor groove near the nucleosome entry site, raising the possibility that Lys methylation influences local histone - DNA interactions [145].

The tight regulation of these enzymes is further exemplified by their dual potential as tumor suppressors or oncogenes, depending on tissue context, driven by expression levels or mutations [139]. One important layer of regulation—potentially acting in a loci-specific manner—is histone cross-talk. Many JMJDs carry additional reader domains that recognize neighboring histone marks and modulate demethylase activity accordingly. For instance, Lys-specific demethylases such as KDM7A-C, KDM2A/B, and KDM5A-D contain PHD domains, while KDM4A-C include both PHD and Tudor domains–histone reader modules that recognize methylated or unmodified histones to control chromatin binding affinity and structural rearrangements, thereby fine-tuning demethylase activity.

KDM5A, which represses transcription by demethylating the activating H3K4me1/2/3 marks, contains a PHD1 domain adjacent to its catalytic core that Longbotham et al. found preferentially binds unmodified H3 tails *in trans*. This interaction enhances demethylase activity through affecting the *K*_m_ - likely through conformational stabilization and/or allosteric changes to strengthen H3K4me-binding [146]. On the other hand, the presence of phosphorylated residues like H3T3 or H3T6 (when present *in trans*) reduces demethylase activity, likely by weakening the interaction between PHD1 and the histone substrate. This inhibition is consistent with a broader model presented by Gatchalian and colleagues where PHD domains disengage from H3K4me3-enriched chromatin during mitosis, when histone phosphorylation is prevalent [147].

In contrast to JMJDs that use accessory domains like PHD fingers to tune substrate preferences, LSD1 takes a different approach. It relies on a specialized catalytic domain architecture to directly sense *in cis* histone cross-talk and modulate demethylase activity. LSD1 forms a physiological complex with the CoREST scaffolding protein (LC complex), functioning mainly as a demethylase. This complex can also associate with HDAC1/2 (forming LHC complex), allowing it to function as a deacetylase [148].

A cluster of polar residues near the LSD1 active site contributes binding energetics and supports the conformational flexibility required for efficient H3K4me1/2 demethylation [149–151]. This architecture allows for nuanced *in cis* cross-talk between H3K4me1/2 and neighboring modifications—including H3K9ac, H3K14ac, H3K18ac, H3T6ph, and H3S10ph—which collectively reorganize intramolecular and intermolecular hydrogen bonding networks in the H3 tail when bound to LSD1, suppressing enzymatic activity [44,152,153].

**Figure 7:**
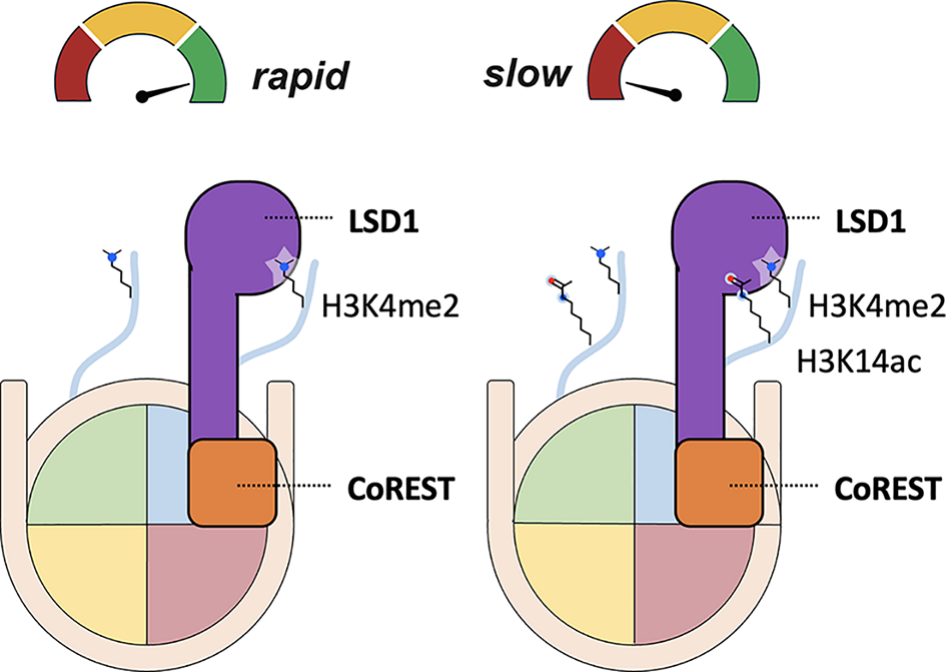
Histone cross-talk regulates the kinetics of LSD1 Left: LSD1-CoREST rapidly demethylates H3K4me2 nucleosomes by directly engaging the modified H3 tail. Right: LSD1-CoREST demethylates H3K4me2-H3K14ac nucleosomes more slowly, as the acetyl group on Lys14 of the same tail suppresses catalytic activity despite having minimal effect on LSD1-CoREST’s binding affinity for the nucleosome.

Among these, H3K4me1/2-H3K14ac cross-talk particularly stands out (Figure 7). This combinatorial mark is a poor substrate for both the LC CoREST demethylase complex [148] and the LHC CoREST deacetylase complex [44,46,154], making it broadly resistant to CoREST-mediated chromatin remodeling. Notably, H3K4me1-K14ac is enriched at cis-regulatory elements of genes involved in cell adhesion and myeloid leukocyte activation in K562 chronic myelogenous leukemia cells, suggesting a possible functional role for this specific cross-talk [148]. It’s also worth noting that H3K14ac inhibits Jhd2–the yeast homolog of KDM5A that targets H3K4me1/2/3. Moreover, H3 tail acetylation is associated with enhanced H3K4 methylation by MLL [114,115]. These results suggest a potentially conserved mechanism of H3K14ac-mediated repression within the demethylases [155]. While this inhibition may occur *in trans* for the KDM5A counterpart, in the case of LSD1, the inhibitory effect of H3K4me1/2-K14ac is specifically potent *in cis* [49], further highlighting the variable sensitivity of histone demethylases to histone cross-talk.

#### Acetyltransferases

The human histone acetyltransferases (HATs) include: HAT1, GCN5 (KAT2A) and its paralog PCAF (KAT2B), MYST family members KAT5-KAT8, and p300/CBP (KAT3A/B) [156]. General Control Non-depressible 5 (GCN5) is a founding member of the GNAT (Gcn5-related N-acetyltransferase) family, which shares a conserved four-motif catalytic core [157]. While following a classic sequential mechanism, GCN5 catalytic efficiency and site specificity are enhanced by its interacting partners Ada2 and Ada3, and its bromodomain and the Sgf29 Tudor domain within SAGA [19,158,159]. GCN5 preferentially acetylates H3K9 and H3K14 in nucleosomes and functions within the SAGA and ATAC coactivator complexes [160].

The KAT3 family (p300/CBP) features a distinct catalytic fold, a bromodomain, and an autoinhibitory TAZ2 domain. Activation of p300 occurs through autoacetylation and binding of transcription factor activation domains, such as from p53 or BRD4-NUT fusions [161]. Unlike other HATs, p300 follows a proposed ping-pong mechanism (instead of sequential mechanism with the formation of a ternary complex) to acetylate multiple sites, including H3 K18 and K27; H2B K5, K11, K12, K15, K16, K20 and K23; and H4K8 [162]. It interacts dynamically with reader proteins and transcription factors to facilitate enhancer and promoter activation [163,164]. p300-mediated H3K27ac can promote H3K4me3 deposition, revealing a directional cross-talk pathway dependent on BRD2, not BRD4 [165].

The MYST family is comprised of TIP60 (KAT5), MOZ/MORF (KAT6A/B), HBO1 (KAT7), and MOF (KAT8). All members have roles in gene regulation with some divergence in specific roles ranging from DNA damage repair and stem cell renewal to development [166,167]. MYST family proteins are more similar to GNATs than p300/CBP, with a sequential catalytic mechanism with the formation of a ternary complex [168]. The MYST HAT domain consists of two β-sheets surrounded by α-helices with an acyl-CoA binding site and conserved catalytic glutamate and cysteine residues [169]. There are many instances of cross-talk among the MYST family: the chromodomain on TIP60 reads H3K9me3 at double-strand breaks and stimulates ATM activation [170], BRPF3 facilitates HBO1 acetylation of H3K14 at replication origins and TSS [171], MOF regulates H4K16ac and was shown to be involved in a trans histone modification pathway involving H3T11 phosphorylation [172].

##### SAGA

The SAGA (Spt–Ada–Gcn5 acetyltransferase) complex is a ∼2 MDa multi-subunit coactivator conserved from yeast to humans. It functions at the interface of transcription initiation, elongation, chromatin remodeling, histone modification, and mRNA export [173,174]. Within SAGA, the HAT module comprised of general control nonderepressible 5 (GCN5), ADA2, ADA3, and Sgf29, acetylates H3 tails, while the DUB module (Ubp8/Sgf11/Sus1/Sgf73 in yeast; USP22/ATXN7L3/ENY2 in humans) removes H2BK120Ub [175]. Bonnet et al. found that H2B ubiquitination is required for proper H3K4 and H3K79 methylation. Thus, the DUB and HAT modules together influence promoter activity and transcription elongation [176]. *In vitro* nucleosome studies by Ringel et al. demonstrate that Sgf29 binding to H3K4me3 enhances the processivity and specificity of GCN5-mediated acetylation, particularly of H3K14 and H3K18. Sgf29 enables preferential acetylation of methylated nucleosomes even in the presence of excess unmodified chromatin—a hallmark of reader-guided substrate discrimination [159]. Moreover, Cieniewicz and colleagues found GCN5’s bromodomain reads acetyl-Lys (e.g., H3K14ac), promoting feed-forward acetylation at adjacent sites. Mutation of the bromodomain diminishes acetylation of downstream lysines like H3K18, demonstrating a reader–writer coupling mechanism [158].

In SAGA, reader-writer cross-talk occurs via Sgf29, where the recognition of H3K4me3 stimulates H3 acetylation. The tandem Tudor domain of Sgf29 reads H3K4me2/3, a hallmark of active promoters. This binding is mediated through a negatively charged pocket that includes aromatic residues (Y238, F264, Y265, D266) and further stabilized by interactions with H3 A1 and R2 [177]. Recognition of H3K4me3 nucleosomes by Sgf29 increases the processivity and efficiency of GCN5-mediated acetylation at multiple H3 lysines, including K9, K14, and K18. This effect is abolished when the Tudor domain is deleted or mutated, confirming that the cross-talk is dependent on methyl-Lys recognition [159]. *In cellulo* studies by Bian et al. and *in vitro* studies by Ringel et al. support that Sgf29-mediated recruitment of the HAT module to H3K4me3-marked promoters promotes dense acetylation of H3 tails, particularly at the +1 nucleosome, reinforcing transcription initiation [159,177].

In addition, bromodomain-mediated feedforward acetylation has been reported by Cieniewicz et al. [158]. GCN5 contains a C-terminal bromodomain that binds acetylated H3K14. This recognition event enhances further acetylation at adjacent lysines (K9, K18, K23, K27, K36), functioning as a cis-histone cross-talk amplifier. Mutation of the bromodomain disrupts processive acetylation, with H3K18ac most strongly affected. This supports a model where H3K14ac not only signals chromatin accessibility but also facilitates sequential acetylation events [158]. The GCN5 bromodomain has also been shown to help HAT activity in a trans nucleosome manner and even when H4K16ac is present, according to Li et al. [178]. In addition, Kim et al. found that GCN5 bromodomain engagement contributes to chromatin recruitment and release of downstream remodeling complexes such as SWI/SNF and RSC, which contain their own bromodomains and recognize GCN5-acetylated lysines [179].

##### P300

The transcriptional coactivator and Lys acetyltransferase p300 (KAT3B) is a central regulator of gene expression that acetylates both histone and non-histone substrates [180]. Dysregulation of p300 can drive oncogenesis, making it an attractive drug target for blocking both its bromodomain and HAT domain [181]. p300 acetylates multiple sites on H2B, H3, and H4 tails—most notably H3K27ac and H3K18ac—marks enriched at active promoters and enhancers [182,183]. Unlike SAGA or NuA4, p300 does not operate as part of a stable, biochemically isolated complex. Instead, it acts as a modular enzymatic scaffold, regulated through dynamic interactions with transcription factors and reader proteins [184]. p300 catalytic activity is autoinhibited by its own TAZ2 domain and activated upon partner binding, autoacetylation, or chromatin recruitment [161].

A direct example of p300-mediated histone cross-talk was uncovered using locus-specific CRISPR/dCas9 epigenome editing by Zhao et al [165]. Targeted installation of H3K27ac at the IL1RN and GRM2 promoters by a dCas9-p300 fusion led to robust enrichment of H3K4me3 at TSS and activated gene expression. In contrast, targeted installation of H3K4me3 via a dCas9-SET1 fusion was insufficient to induce H3K27ac or transcription. These results suggest a unidirectional cross-talk mechanism in which H3K27ac acts upstream of H3K4me3 at active promoters [165]. Mechanistically, this effect required BRD2, not BRD4, as a reader of H3K27ac to facilitate recruitment or stabilization of the H3K4 methylation machinery. Pharmacologic inhibition of BRD proteins (JQ1 small molecule inhibitor) blocked both H3K4me3 deposition and transcriptional activation in response to p300-mediated H3K27ac, further confirming reader-dependent propagation of active histone marks [165].

Moreover, p300 harbors its own bromodomain (BrD), which recognizes acetyl-lysine and can contribute to substrate selection. Work by Zucconi and colleagues has shown that cross-talk between p300’s BrD and its catalytic HAT domain regulates access to histone tails and may promote multi-site acetylation [185]. Additionally, Yu et al. found that activation of p300's catalytic domain is tightly regulated by the TAZ2 domain, which can autoinhibit HAT activity until displaced by α-helical transcription factor domains such as those from p53, BRD4, or NUT [161]. Biochemical studies by Kikuchi et al. have suggested that the bromodomain of p300 can bind to H4 acetylation sites and this triggers H2B acetylation in the same nucleosomes [186].

##### MORF

Transcriptional coactivator monocytic leukemia zinc-finger protein-related factor (MORF) complex was shown by Klein et al. to be a global reader of H3K4 acylation via its double plant homeodomain finger (DPF) on its catalytic subunit [187]. MORF and its homolog, Monocytic leukemia zinc finger protein (MOZ) also contain three adapter proteins, bromodomain and PHD finger-containing protein (BRPF1/2/3), Inhibitor of growth protein 5 (ING5), and hEAF6. The full complex contains a bromodomain, a PWWP domain, and several PHD fingers; only hEAF6 doesn't contain a reader domain [169]. Germline mutations cause developmental syndromes, and dysregulation or translocations contribute to leukemias, which spurs interest in inhibitors now explored as anticancer strategies [188]. The human MORF complex showed a preference for H3 peptides with H3K14 butyrylation (with a *K*_d_ of 1.2 μM, followed by acetylation, 2-hydroxyisobutyrylation, then succinylation). An H3 acylation profile was performed with six HATS and uncovered that there are differences in the writers’ longer acyl group preference among HATS, with GCN5/PCAF able to propionylate and butyrylate, whereas CBP showed a preference for propionylation and MOF for butyrylation [187]. Klein et al. resolved a crystal structure of the MORF DPF domain and a H3K14cr peptide and found that H3K23 acetylation is promoted by DPF binding to H3K14ac, which activates transcription [189]. Qiu et al. found that MOZ also regulates HOXA9 transcription through reading combinatorial H3R and H3K14ac [190].

#### Histone deacetylases

Histone deacetylases (HDACs) remove acetyl groups from histone proteins, leading to a more compact chromatin structure that reduces gene expression. HDACs are categorized into four classes based on their sequences and the type of cofactors that they use [191,192]. Given that altered levels of HDACs significantly affect the development of human diseases such as cancer, inflammatory diseases, and neurodegenerative diseases, HDACs are targets for multiple FDA-approved chemotherapeutics [193]. Class I (HDAC1, 2, 3, and 8), Class IIa (HDAC4, 5, 7, and 9), and Class IIb (HDAC6 and 10) are Zn(II)-dependent hydrolases. Class III HDACs (Sirtuins) use NAD+ as a cofactor, and subsequently produce *O*-acetyl ribose and nicotinamide as byproducts of their catalytic activities [191,192]. Recent studies have shown that HDAC-mediated deacylation both regulates and is regulated by other histone PTMs. In the case of Class I HDAC complexes it appears to be the various scaffolding proteins [Corepressor of REST (CoREST), Metastasis-associated protein 1 (MTA1), Sin3, and Mitotic deacetylase associated SANT domain protein (MiDEAS)] among others that play a crucial role in histone PTM cross-talk.

##### CoREST complex

The CoREST complex that comprises lysine-specific demethylase 1 (LSD1), HDAC1/2, and CoREST scaffolding proteins (LSD1–HDAC1/2–CoREST; LHC) removes both acetyl and methyl groups through the activities of HDAC1/2 and LSD1 [44,194]. Wu et al. have revealed that the LHC complex slowly catalyzes demethylation, while it efficiently removes acetyl groups from nucleosomes, with a preference for H3K9ac, followed by H3K18ac and H3K14ac. In LHC/CoREST *in vitro* assays, the deacetylation activities in the CoREST complex precede demethylation, and particularly, the acetylation at H3K14 significantly affects LSD1-induced demethylation [44].

##### NuRD complex

In addition to the CoREST complex, deacetylation activities of the nucleosome remodeling and deacetylase repressor (NuRD) enzyme complex are reduced by the methylation of H3K4 [195]. The NuRD complex is composed of HDAC1/2, MTA1/2/3, methyl-DNA binding domain (MBD2/3), chromodomain- helicase-DNA-binding protein (CHD3/4), P66a/b also known as GATAD2A/B (GATA Zinc Finger Domain Containing 2A/B), and RBBP4/7 [196]. Zegerman et al. found that the two plant homeobox domain (PHD) of CHD4 preferentially bind to unmodified H3K4 and H3K9me3. Mansfield et al. determined that the interaction with H3K4 depends on hydrogen bonds between the PHD and the lysine side chain, which trimethylation of H3K4 disrupts, leading to reduced deacetylation efficiency of the enzyme complex [195,197].

##### Rpd3S complex

The small histone deacetylase reduced potassium dependency 3 (Rpd3S) complex, composed of Rpd3, Sin3, Ume1, Rco1, and Eaf3 subunits, has recently attracted increasing research interest. It has been shown that histone H3K36 trimethylation serves as a regulator of the deacetylation activity of the Rpd3S complex towards histone H3 and H4 proteins [198–203]. Methylation of H3K36 by Set2 methyltransferase induces the recruitment of the Rpd3S complex to nucleosomes, where it initiates histone deacetylation and inhibits intragenic transcription [204]. Among the subunits of the Rpd3S complex, the chromodomain (CHD) of Eaf3 and the PHD domain of Rco1 are required for the recognition of H3K36 methylation mark and unmodified H3 tail, respectively [199,205]. In detail, the deletion of the CHD of Eaf3 and the PHD domain of Rco1 in the Rpd3S complex does not affect the integrity of Rpd3S; however, the deletion of the CHD of Eaf3 reduces the binding affinity to nucleosomes and leads to loss of binding specificity to the H3K36-methylated nucleosomes. The deletion of the PHD of Rco1 in the Rpd3S complex disrupts Rpd3S interaction with nucleosomes, indicating a distinct role of Eaf3 and Rco1 in interactions with nucleosomes [199]. In addition, Rpd3S complex binds to nucleosomes with linker DNA and further prefers di-nucleosomes [199,200].

**Figure 8:**
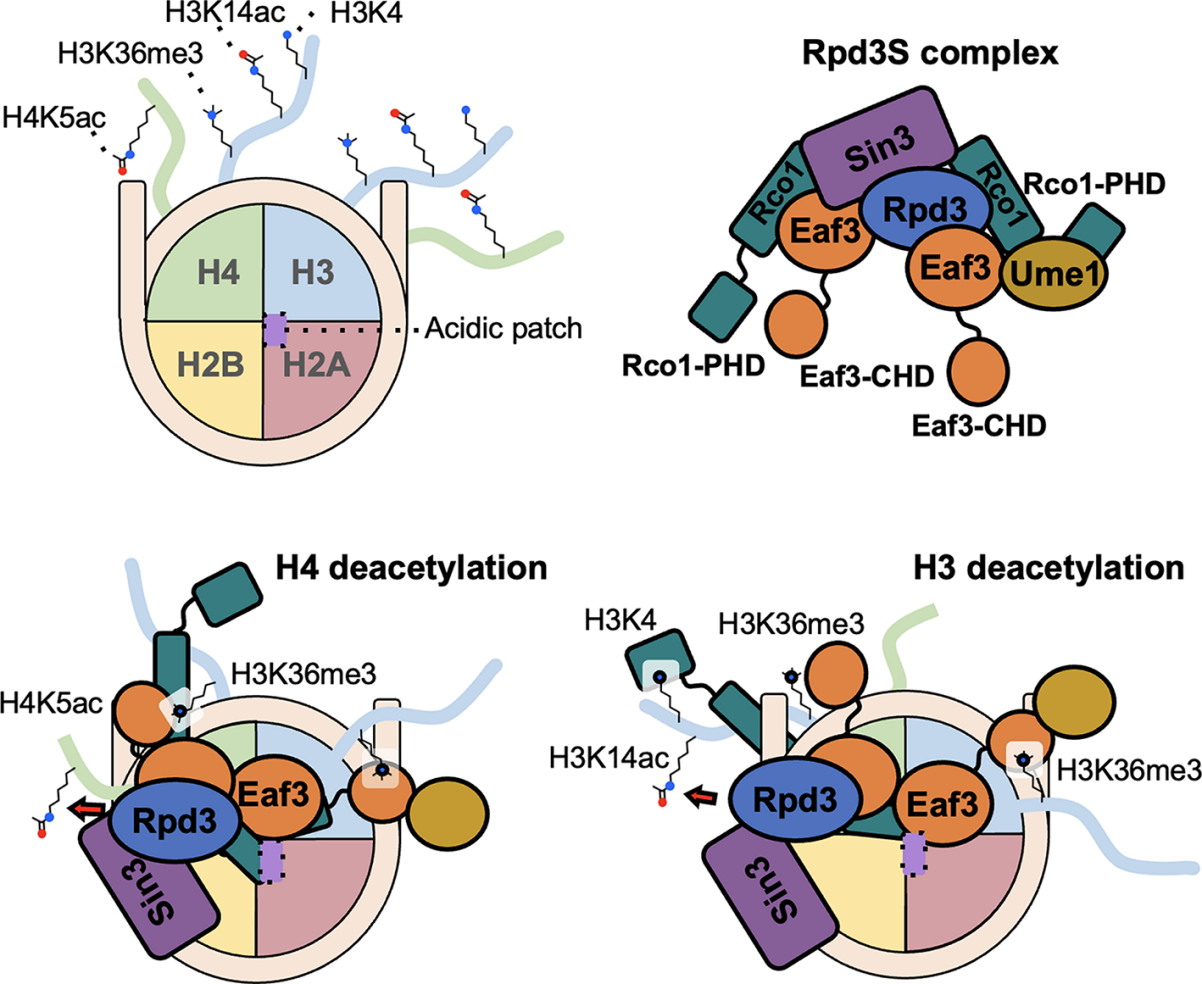
Regulation of histone deacetylation by the Rpd3S complex Top: Composition of the Rpd3S complex and its targets and binding sites in nucleosomes. Bottom: Predicted interactions between the Rpd3S complex and nucleosome for H4 and H3 deacetylation, respectively. Rpd3S shows rapid deacetylation of H3 and H4 tails except for H3K9ac.

Recent structural studies using Cryo-EM have provided further insight into the molecular mechanisms for how the Rpd3S complex recognizes nucleosomes and performs its deacetylation activity (Figure 8). The Rpd3S complex contains single copies of Rpd3, Sin3, Ume1, and two copies of Rco1 and Eaf3 [198,200–203]. The structural model of the Rpd3S–di-nucleosome complex, suggested as the preferred binding mode, shows that the second copy of Eaf3 and Rco1 can be located next to the neighboring nucleosome; however, it is challenging to acquire a high-resolution structure of the di-nucleosome complex due to its presumed structural flexibility [201]. Thus, high-resolution structural models have primarily been obtained using mono-nucleosomes [198,200–203]. Guan et al. presented two engagement models with the Rpd3S–H3K36me3 nucleosome complex (“close” and “loose”), distinguished by the interactions between the MID domain of Rco1-A and the linker DNA [198]. Both models show the recognition of the two CHDs of Eaf3 with H3K36me3 at the DNA super-helical location (SHL) +1 and +7, and positions of the catalytic center of Rpd3 near the H4 tail for deacetylation [198]. Markert et al. present the interaction of the Rpd3S complex with the H2A–H2B acidic patch region, suggesting the acidic patch region-mediated recruitment of the Rpd3S complex to nucleosomes for the promotion of its deacetylation activity (Figure 8) [202]. Structural investigations from Dong et al. also suggest that the Rpd3S complex can tighten the extranucleosomal DNA after deacetylation [203]. The structure of the Rpd3S–H3K36me3 nucleosome complex obtained from Li et al. indicates the recruitment of the PHD1 domain of Rco1 to the *N*-terminus of H3, which induces binding of the H3 tail to the Rpd3S catalytic center [201]. The formation of salt bridges, particularly between amino acids in the *N*-terminal region of H3 and the PHD1 domain of Rco1, suggests the reason for the reduced binding of the Rpd3S complex to nucleosomes when H3K4 is trimethylated [201]. Wang et al. suggested how H3K14 or H3/H4 tails can be accommodated in the Rpd3S catalytic center based on their three distinct structural models of the Rpd3S complex with nucleosomes isolated from HEK293T cells [206].

Methylation at H3K36 enhances the deacetylation activity of the Rpd3S complex toward acetylated H3 and H4 tails [200]. Notably, Huh et al. found that the Rpd3S complex exhibited more enzymatic activity on H3K36me3 di-nucleosomes than on mono-nucleosomes [200]. The enzymatic activities of the Rpd3S complex towards the acetylated lysines at H3 (i.e., K9ac, K14ac, K18ac, K23ac, K27ac) and H4 (i.e., K5ac, K8ac, K12ac, K16ac) have revealed that it effectively deacetylates these Lys residues except for H3K9ac *in vitro* and *in vivo* [198,201]. This can be attributed to the binding of unmodified H3K4 to the PHD1 domain of Rco1, which in turn restricts the positioning of H3K9 into the catalytic center of the Rpd3S complex [198,201]. Together, all these investigations collectively demonstrate the multivalent interactions between the Rpd3S complex and nucleosomes, indicating how the subunits of the Rpd3S complex cooperatively work to recognize different histone PTM marks for its enzymatic function.

With its preference for unmodified H3K4 and H3K36me3, Rpd3S is likely most active in the gene body where this modification state is prevalent; however, HDACs also direct deactivation of the transcription start site, defined by a different set of H3 modifications. At the transcription start site, the activity of coactivators like p300 and CBP has been described as an “acetyl spray,” targeting histones and components of the transcriptional machinery, creating a hyperacetylated environment. Nonetheless, the half-lives of many of these histone acetylation sites are in the minutes, suggesting countervailing HDAC activity [183].

##### MiDAC complex

Comparing HDAC site selectivity on monoacetylated nucleosomes versus hyperacetylated nucleosomes (acetylated at H3K9, K14, K18, K23, and K27) revealed that hyperacetylation reshapes HDAC site preference [49]. The mitotic deacetylase complex (MiDAC), which contains HDAC1/2, MIDEAS, and DNTTIP1, deacetylates H3K27ac approximately 4-fold more slowly when H3K27ac is the only modification present on the nucleosome. In contrast, H3K27ac deacetylation is more rapid in the presence of additional acetylations on the H3 tail. This enhancement (∼3-fold) persisted in asymmetric nucleosomes in which H3K27ac was present on one H3 tail, while the opposing H3 tail carried acetylation at H3K9, K14, K18, and K23 (*in trans*). These findings indicate that MiDAC-mediated histone deacetylation is driven by a processive mechanism at the level of nucleosomes rather than by the increased histone tail mobility resulting from hyperacetylation [12]. Consistent with this model, MiDAC primarily exists as a tetrameric complex comprising four catalytic HDAC1/2 subunits arranged in a cruciform architecture, suggesting MiDAC’s multivalent binding modes toward hyperacetylated nucleosomes and cross-talk between H3 tails [207].

##### Sirtuins

Members of the NAD^+^-dependent Class III HDACs, Sirtuins, also exhibit histone cross-talk. Like Class I HDACs, Sirtuin selectivity is influenced by combinations of modifications that characterize active TSS including modifications like H3K4me3 and acylation of H3K9, K14, K18, K23 and K27. Using singly acetylated nucleosome substrates, SIRT2 was found to deacetylate H3K27 2- to 3-fold faster than any of K9, K14, K18 or K23. Conversely, with nucleosomes in which H3 is acetylated at all of K9, K14, K18, K23 and K27, SIRT2 was found to shift selectivity and deacetylate K18 2- to 3-fold faster than any other site, including K27 [49]. Using asymmetric nucleosomes, it was confirmed that the cross-talk between K18ac or K23ac and other acetylations on the H3 tail only occurs on the same copy of H3 (*in cis*). The opposite was observed to be the case for K27ac, which was observed to undergo slower deacetylation regardless of whether additional acetylations were *in cis* or on the second copy of H3 (*in trans*).

The same multiply acetylated nucleosome approach was used to evaluate SIRT6, revealing similarly slowed (2-fold) deacetylation of K27. As with SIRT2, SIRT6 slowing was independent of which copy of histone H3 carried additional acetylations. In spite of this K27-specific slowdown, no changes in the site-selectivity of SIRT6 were observed overall [49,145]. From this, it would be tempting to conclude that highly acetylated chromatin regions buffer against removal of K27ac by Sirtuins. Stable occupancy of the DNA minor groove by H3K4 in multiple structures of nucleosome-bound SIRT6 led to the hypothesis that H3K4me3 might serve a similar sheltering function, however independent evaluations found no significant effect on deacetylation of H3K9ac by SIRT6 [145,208,209].

Rather than directly interacting with histone tails, SIRT1 regulates the acetylation of histone methyltransferases and their catalytic efficiency, thereby altering the methylation level of histones [210,211]. For example, suppressor of variegation 3–9 homologue 1 (SUV39H1) methyltransferase loses its H3K9 methylation activity in SIRT1 knockout mouse embryonic fibroblast cells [210]. It has been identified that the methylation activity of SUV39H1 relies on the presence of the *N*-terminal region of SIRT [210]. Studies by Vaquero et al. using HDAC inhibitors (i.e., trichostatin A and nicotinamide) and mutagenesis of SUV39H1 reveal that the deacetylation of K266 in the catalytic SET domain of SUV39H1 by SIRT1 is also critical to promote the enzymatic activity of SUV39H1 [210]. Moreover, recent studies in circadian control by Aguilar-Arnal et al. have shown that SIRT1 deacetylates the histone methyltransferase Mixed-Lineage Leukemia 1 (MLL1) to regulate its methylation activity [211]. Mass profiling and mutagenesis studies suggest that K1130 and K1133 within MLL are the target sites of SIRT1 [211]. Different from the role of SIRT1 in SUV39H1, SIRT1 downregulates the level of H3K4me3 mediated by MLL1 [211]. In the presence of NAD^+^, activated SIRT1 deacetylates MLL, in turn reducing the level of H3K4me3 and repressing the circadian gene expression [211].

## Conclusion

This article summarizes some exemplary mechanisms by which one histone modification can influence another on the same nucleosome or on the same histone tail. Such effects are seen with writer enzymes such as acetyltransferases and methyltransferases as well as eraser enzymes, including deacetylases and demethylases. There is a growing number of chemical, protein engineering, and analytical methods which are increasing the feasibility of studying asymmetric nucleosomes and modification cross-talk. Given the explosion in the number of histone PTM types identified, however, the molecular complexity for analysis continues to grow. More extensive functional and structural studies are needed to understand the significance of cross-talk in healthy and disease states. As new epigenetic therapies make their way to the clinic, understanding the full range of combinatorial histone modification effects will continue to be important in predicting pharmacological response and potential toxicity as well as understanding pathophysiological mechanisms.

## Open Access

Open access for this article was enabled by the participation of Ohio State University in an all-inclusive *Read & Publish* agreement with Portland Press and the Biochemical Society under an agreement with the Big Ten Alliance.

## Abbreviations

CHD: chromodomain
EPL: expressed protein ligation
MLL: Mixed-Lineage Leukemia 1
NCL: native chemical ligation
PTM: post-translational modification
SHL: super-helical location
SML: Sortase-mediated ligation
UBL: ubiquitin-like modifier
